# The Effect of Criticism on Functional Brain Connectivity and Associations with Neuroticism

**DOI:** 10.1371/journal.pone.0069606

**Published:** 2013-07-26

**Authors:** Michelle Nadine Servaas, Harriëtte Riese, Remco Jan Renken, Jan-Bernard Cornelis Marsman, Johan Lambregs, Johan Ormel, André Aleman

**Affiliations:** 1 Neuroimaging Center, Department of Neuroscience, University Medical Center Groningen/University of Groningen, Groningen, The Netherlands; 2 Interdisciplinary Center for Psychopathology and Emotion Regulation, University Medical Center Groningen/University of Groningen, Groningen, The Netherlands; 3 Department of Epidemiology, University Medical Center Groningen/University of Groningen, Groningen, The Netherlands; 4 Department of Psychology, University of Groningen, Groningen, The Netherlands; Hangzhou Normal University, China

## Abstract

Neuroticism is a robust personality trait that constitutes a risk factor for psychopathology, especially anxiety disorders and depression. High neurotic individuals tend to be more self-critical and are overly sensitive to criticism by others. Hence, we used a novel resting-state paradigm to investigate the effect of criticism on functional brain connectivity and associations with neuroticism. Forty-eight participants completed the NEO Personality Inventory Revised (NEO-PI-R) to assess neuroticism. Next, we recorded resting state functional magnetic resonance imaging (rsfMRI) during two sessions. We manipulated the second session before scanning by presenting three standardized critical remarks through headphones, in which the subject was urged to please lie still in the scanner. A seed-based functional connectivity method and subsequent clustering were used to analyse the resting state data. Based on the reviewed literature related to criticism, we selected brain regions associated with self-reflective processing and stress-regulation as regions of interest. The findings showed enhanced functional connectivity between the clustered seed regions and brain areas involved in emotion processing and social cognition during the processing of criticism. Concurrently, functional connectivity was reduced between these clusters and brain structures related to the default mode network and higher-order cognitive control. Furthermore, individuals scoring higher on neuroticism showed altered functional connectivity between the clustered seed regions and brain areas involved in the appraisal, expression and regulation of negative emotions. These results may suggest that the criticized person is attempting to understand the beliefs, perceptions and feelings of the critic in order to facilitate flexible and adaptive social behavior. Furthermore, multiple aspects of emotion processing were found to be affected in individuals scoring higher on neuroticism during the processing of criticism, which may increase their sensitivity to negative social-evaluation.

## Introduction

Most people like to hear that they are performing well, both in their personal as well as their professional life. Inevitably, people's behavior is sometimes negatively judged or criticized by others. Individual differences in stress reactivity play an important role in the way people deal with criticism and other forms of negative social-evaluation [1]. How people cope with stress is determined -among other factors- by their personality. A personality trait that has specifically been associated with stress sensitivity is neuroticism [2]–[5]. Neuroticism is one of the Big Five dimensions of personality and represents a robust trait that has been replicated many times in various studies [6]. High neurotic individuals express heightened emotional reactivity, especially to negative events [7] and are more prone to develop psychiatric disorders, such as depression and anxiety disorders [8]. Moreover, these individuals tend to be more self-critical [9] and are overly sensitive to criticism by others [5].

To our knowledge, the interaction between criticism and neuroticism has not previously been studied using functional magnetic resonance imaging (fMRI). Studies investigating the effect of criticism on brain function are limited as well. However, it has been shown that listening to criticism activates brain areas involved in the cognitive control over negative emotions and self-referential processing [10]. Furthermore, differential processing of criticism has been related to several psychiatric disorders. For instance, Blair et al. (2008) found that patients diagnosed with generalized social phobia (GSP) showed increased activation in the medial prefrontal cortex and the amygdala, as well as enhanced functional connectivity between these two areas in response to negative comments referring to themselves in comparison to healthy controls [11]. In addition, Hooley et al. (2009) showed that even though patients were remitted from depression, their brain functioning was still altered in response to hearing critical comments made by their own mothers compared to healthy controls [12]. Moreover, previous research has shown that formerly depressive patients are more likely to relapse, when they perceive their significant family members as being critical of them (‘perceived criticism’). This has also been replicated in other patients samples, including anxiety disorders, schizophrenia and substance abuse disorders [13]. Individuals that score high on perceived criticism show increased limbic reactivity and decreased cognitive regulatory prefrontal activity during the processing of criticism [13].

One may conclude that criticism is a clinically relevant concept and that it is important to identify and map its underlying neurobiological mechanisms. A new challenge would be to investigate the concept of criticism in a setting, where comments are applicable to the individuals' current situation and his or her corresponding behaviour. However, related literature on psychosocial stress has taught us that it proved to be a challenge to create a task paradigm within (i) the neuroimaging environment that is (ii) able to reliably induce a stress response and (iii) has a naturalistic character [14]. A meta-analysis on changes in cortisol -an indicator of the stress response- showed that stress could be elicited by motivated performance tasks, which contain elements of social evaluation (e.g. an evaluative audience is present) and uncontrollability (e.g. false feedback) [1]. However, a limitation of motivated performance tasks is that components related to challenge and achievement play a prominent role, which overshadow the effect of negative social-evaluation [15]. Interpersonal stressor paradigms overcome this limitation but are still strictly virtual simulations of social situations; for example participants are deceived into believing that they are excluded from an online ball-tossing game [16].

To surpass abovementioned drawbacks, we recorded resting state fMRI (rsfMRI) during a newly constructed paradigm in which criticism on the participants' behaviour was applicable to the current situation. The elements (negative) social-evaluation and uncontrollability, shown to be important in eliciting a stress response [1], were incorporated in the paradigm. We presented participants with three standardized critical remarks through headphones, in which the investigator urged the participant to please lie still in the scanner (independent of whether they were lying still or not). The requests were conferred with an increasingly agitated tone. Furthermore, participants were made aware before they went into the scanner that both the investigator and MRI laboratory technician were monitoring them during the experiment. rsfMRI provides an excellent tool to investigate undirected behaviour in participants; it has been shown that intrinsic activity can be modulated by exogenous factors [17] but is task independent in principle.

The aim of the current study was twofold. First, we investigated which functional connectivity patterns underlie the processing of criticism, using seed-based functional connectivity and subsequent cluster analysis. Based on the reviewed literature related to criticism [1], [10]–[12], we selected regions of interest associated with self-reflective processing: frontal, temporal, parietal and cortical midline structures (Table 1) [18] and stress regulation: the amygdala and hippocampus [19]. We hypothesized enhanced functional connectivity between selected seed regions and brain areas involved during the processing of emotions and social interaction. Second, we investigated whether neuroticism explained variance within functional connectivity patterns related to criticism. We hypothesized altered functional connectivity between selected seed regions and brain areas related to emotion regulation in individuals scoring higher on neuroticism [5]. The former as well as the latter hypothesis were confirmed.

**Table 1:** Seed regions associated with self-reflective processing.

Seed region	Brodmann area	Coordinates		
		x	y	z
Anterior cingulate gyrus	32	−2	42	12
Cuneus	18/23	−4	−64	24
Left inferior frontal gyrus, orbital part	47	−38	22	−12
Left insula	48	−38	16	−8
Left superior frontal gyrus	9	−10	44	32
Left temporal pole	38	−40	24	−20
Superior medial frontal gyrus	9	−12	45	34
Superior medial frontal gyrus	10	−2	56	8
Posterior cingulate gyrus/precuneus	23/30	−2	−60	20

Seed regions based on a meta-analysis of neuroimaging studies investigating self-reflection. Contrast (self > baseline) (van der Meer, et al., 2010).

## Materials and Methods

### Participants

Forty-eight healthy Dutch participants (32 women, mean age 20.78±SD 2.45; 16 men, mean age 20.63± SD 2.16, age range: 18–27) were recruited from the University of Groningen. Participants were screened for exclusion criteria using a self-report checklist, comprising the following criteria (1) a history of seizure or head injury, (2) a life time diagnosis of psychiatric and/or neurological disorders, (3) a life time diagnosis of psychiatric disorders in first degree relatives of the participant, (4) the use of medication that can influence test results, (5) visual or auditory problems that cannot be corrected, (6) MRI incompatible implants or tattoos, (7) claustrophobia, (8) suspected or confirmed pregnancy.

### Ethics statement

The Medical Ethical Committee of the University Medical Center Groningen approved the experimental protocol and written informed consent was obtained from all participants prior to participation. The study was conducted in accordance with the Declaration of Helsinki.

### NEO (Neuroticism, Extraversion, Openness) Personality Inventory Revised

The NEO-PI-R [2] is based on the Five-Factor Model (FFM) of personality [20] and consists of 240 items, which assess the following five domains: Neuroticism, Extraversion, Openness, Agreeableness, and Conscientiousness. The psychometric properties of the NEO-PI-R can be considered good. Cronbach's alpha ranges from 0.86 to 0.92 for the domain scales of the Dutch version of the NEO-PI-R [21].

### Stress manipulation

rsfMRI data were recorded during two sessions, each lasting five minutes. Participants were instructed to close their eyes and to not fall asleep. The first session consisted of scanning a standard resting state (standard session). The second session was manipulated before scanning by presenting three standardized critical remarks through headphones (criticism session). The general request addressed to the participant was to please lie still in the scanner. Participants were able to respond after each remark. The first remark: “It is important that you lie still” (neutral tone) was presented at time zero. The second remark: “[harrumph] Could you please lie still now for a moment” (slightly agitated tone) was presented after the scan preparation. The third remark: “Lie still now please” (agitated tone) was presented after the second remark, depending on the length of the participants' reaction to the second remark. The scan was proceeded, after the corresponding reaction to the third remark. The remarks were recorded by the investigator (J.L., male voice). In this way, participants were criticized by the person, who led the experiment. Furthermore, participants were introduced to the MRI laboratory technician, before they went into the scanner. Participants were made aware that both the investigator and MRI laboratory technician were monitoring them during the experiment. After the scanning session, participants were debriefed and informed that the repeated requests to lie still were part of the experiment. The order of the sessions was kept constant; the criticism session always followed the standard session.

To validate our stimuli, a pilot study was conducted to demonstrate that the tone of the three critical remarks was indeed perceived as increasingly agitated and that the receipt of the critical remarks was indeed experienced as negative, stressful and arousing. The results showed that the critical remarks were ranked as expected (remark 1 as least agitating and remark 3 as most agitating). Furthermore, positive affect significantly decreased (T_(9)_ = 2.85, p<0.05) after the presentation of the critical remarks, while negative affect significantly increased (T_(9)_ = −4.59, p<0.05) (see 1. Stimulus pilot in File S1 for a full description of the pilot study).

### Image acquisition

A 3 Tesla Phillips Intera scanner (Phillips Medical Systems, Best, the Netherlands), equipped with an 8-channel SENSE head coil, was used to acquire the images. A high-resolution T1-weighted 3D structural image was obtained using fast-field echo (FFE) for anatomical reference (160 slices; TR: 25 ms; TE: 25 ms; FOV: 256×204; 256×204 matrix; voxel size: 1×1×1 mm). Functional images were acquired by T2*-weighted gradient echo planar imaging (EPI) sequences. The criticism session comprised 150 volumes in 40 axial-slices (TR: 2000 ms; TE: 25 ms; FOV: 210×210; 64×66 matrix; voxel size: 3.2×3.2×2.5 mm). The standard session comprised 200 volumes – only the first 150 volumes were used for analysis – in 43 axial-slices (TR: 2290 ms; TE: 28 ms; FOV: 220×220; 64×61 matrix; voxel size: 3.44×3.44×3 mm). Slices were acquired in an interleaved manner and oriented parallel to the AC-PC plane without gap.

### Image analysis

Image processing and statistical analyses were performed using SPM8 (http://www.fil.ion.ucl.ac.uk), implemented in Matlab 7.8.0 (The Mathworks Inc.). The images were corrected for slice timing and realigned using rigid body transformations. After realignment, the mean image was coregistered to the anatomical T1 image. Subsequently, images were spatially normalized to common stereotactic space (MNI T1-template) and resampled to a voxel size of 2×2×2 mm. Lastly, smoothing was applied using a 6 mm kernel full-width at half maximum (FWHM).

Next, a series of preprocessing steps specific to rsfMRI analysis were performed. First, regression of several nuisance variables was applied to remove sources of spurious variance, comprising six rigid body head motion parameters, the global signal, white matter signal and cerebrospinal fluid (CSF) signal. In order to obtain the last two signals, we performed segmentation to create two separate masks and extracted the first eigenvariate from the time series of the included voxels. In addition, the first temporal derivatives of abovementioned nuisance variables were removed. Second, temporal band-pass filtering was applied to detrend the signal and to retain frequencies between 0.008 – 0.08Hz [22].

Subsequently, a seed-based functional connectivity method was used to analyse the data with a General Linear Model (GLM) [23]. A total of thirteen seed regions were defined based on the following criteria: (i) nine seed regions associated with self-reflective processing were based on a meta-analysis of neuroimaging studies investigating self-reflection [18] (see Table 1) (ii) the bilateral amygdala and hippocampus were selected as seed regions based on a review on stress regulation in the central nervous system [19]. Next, a sphere (radius of 6 mm) was created with Marsbar [24] around the nine center coordinates, which were reported for the contrast (self > baseline) in the meta-analysis on self-reflection. The center coordinates reflect voxels with a maximum score in clusters of activation that are reported in a certain percentage of the studies, included in the meta-analysis [18]. The seed regions consisted of 123 voxels and had a volume of 984 mm^3^. With regard to the amygdala and hippocampus, seed regions were constructed using the WFU Pickatlas. Accordingly, the first eigenvariate was extracted from the time series of the voxels in the thirteen specified seed regions per subject for the two sessions. This resulted in twenty-six eigenvariate time courses for every subject, thirteen for the standard session and thirteen for the criticism session. The eigenvariate time courses were added as a regressor at first level per subject for the two sessions separately and the betas were subtracted from each other (criticism > standard). The resulting contrast images were entered in a second level random effect analysis.

For every seed region, a design was built on second level that consisted of two factors: subject and gender. Gender was entered as a factor of no interest in the model because a gender difference was found in neuroticism scores (see the Results section, Neuroticism scores). Hence, neuroticism scores were centered separately for women and men and were entered as a regressor of interest in the model. Differences between the two sessions as well as interactions with neuroticism (positive as well as negative correlations) were investigated. Results were corrected on FWE cluster level (cluster extent, k>20) with an initial threshold of p<0.001 uncorrected.

### Cluster analysis

In order to facilitate interpretation of the results, abovementioned connectivity maps, i.e (criticism > standard) for each of the thirteen seed regions, were clustered into a number of networks. First, the connectivity maps were averaged across subjects and concatenated. This resulted in a two dimensional matrix (D), where rows represented the seed regions and columns the voxels. Second, the number of clusters present in the data was estimated by creating Cattell's screeplot [25] and a maximum profile log-likelihood [26] based on the eigenvalues of the covariance matrix of D. Both methods revealed a four-component solution (see 2. Clustering analysis, Figure S1 in File S1). Third, fuzzy c-means (FCM) clustering was applied to matrix D to group the selected seed regions based on their functional connectivity pattern in four clusters [27], [28]. The same four-component solution (see 2. Clustering analysis, Figure S2a in File S1) and cluster partition (see 2. Clustering analysis, Figure S2b in File S1) were found, when the cluster analysis was performed on the connectivity maps resulting from the contrast (criticism > standard × neuroticism).

## Results

### Neuroticism scores

The mean neuroticism score across the whole sample was 138.75± SD 20.53 and was consistent with the mean reference value mentioned in the NEO-manual [21] for the neuroticism domain within a student sample (research-context, mean 138.4± SD 21.5). Furthermore, a gender difference was found for neuroticism (F_(1,46)_ = 8.55, p<0.05). On average, women had higher scores on neuroticism than men (women: mean 144.44± SD 17.72; men: mean 127.38± SD 21.57) (NEO manual, students, research-context, women: mean 143.6± SD 21.0; men: mean 132.8± SD 20.6).

### Cluster analysis

**Figure 1:**
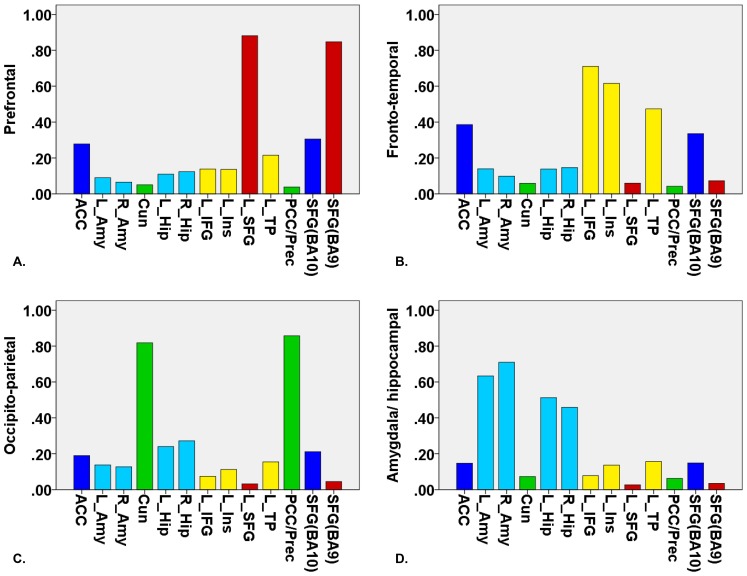
Four clusters were found using fuzzy c-means clustering for the contrast (criticism > standard): (A) prefrontal cluster (red bars), (B) fronto-temporal cluster (yellow bars), (C) occipito-parietal cluster (green bars) and (D) amygdala/hippocampal cluster (light blue bars). The seed regions anterior cingulate cortex and SFG(BA10) are depicted in dark blue. On the x-axis, the different seed regions can be found in alphabetical order. On the y-axis, membership degrees are continuously expressed as proximities to a cluster centroid, containing values between 0 and 1. ACC, anterior cingulate cortex; L_Amy, left amygdala; R_Amy, right amygdala; Cun, cuneus; L_Hip, left hippocampus; R_Hip, right hippocampus; L_IFG, left inferior frontal gyrus; L_Ins, left insula; L_SFG, left superior frontal gyrus; L_TP, left temporal pole; PCC/Prec, posterior cingulate cortex/precuneus; SFG(BA10), superior frontal gyrus (BA10); SFG(BA9), superior frontal gyrus (BA9).

**Figure 2:**
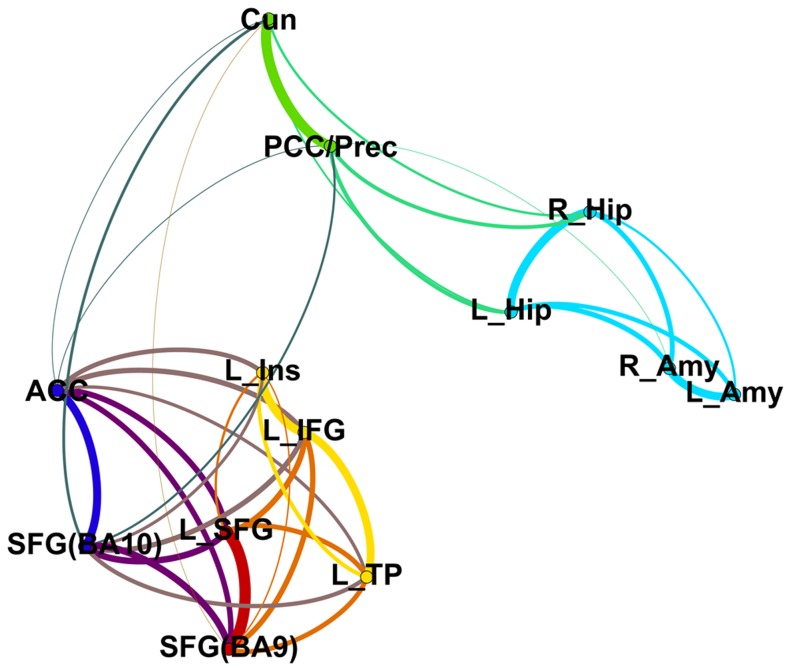
Visualization of correlations between the seed regions based on their functional connectivity pattern. Gephi (0.8.1 – beta) was used to draw the graph. The following colors indicate the cluster to which a specific seed region belongs based on the fuzzy c-means clustering approach: the prefrontal cluster (red), the fronto-temporal cluster (yellow), the occipito-parietal cluster (green) and the amygdala/hippocampal cluster (light blue). The seed regions anterior cingulate cortex and SFG(BA10) are depicted in dark blue. The edges between the nodes have a mixed color. The thickness of the edges represents the strength of the correlation between the seed regions based on their functional connectivity pattern. ACC, anterior cingulate cortex; L_Amy, left amygdala; R_Amy, right amygdala; Cun, cuneus; L_Hip, left hippocampus; R_Hip, right hippocampus; L_IFG, left inferior frontal gyrus; L_Ins, left insula; L_SFG, left superior frontal gyrus; L_TP, left temporal pole; PCC/Prec, posterior cingulate cortex/precuneus; SFG(BA10), superior frontal gyrus (BA10); SFG(BA9), superior frontal gyrus (BA9).

The eigenvalues revealed a four-component solution (see 2. Clustering analysis, Figure S1 in File S1) and therefore, FCM clustering was applied to find four clusters. The first cluster consisted of functional connectivity patterns associated with two seed regions positioned in the prefrontal cortex; the superior frontal gyrus (BA9) and left superior frontal gyrus (prefrontal cluster). The second cluster comprised functional connectivity patterns related to three seed regions located in the fronto-temporal cortex; the left inferior frontal gyrus (orbital part), left insula and left temporal pole (fronto-temporal cluster). The third cluster consisted of functional connectivity patterns associated with two seed regions sited in the occipito-parietal cortex; the posterior cingulate gyrus/precuneus and cuneus (occipito-parietal cluster). The fourth cluster comprised functional connectivity patterns related to four subcortical seed regions: left and right amygdala and hippocampus (amygdala/hippocampal cluster). The seed regions anterior cingulate gyrus and superior frontal gyrus (BA10) loaded on both the first cluster as well as the second cluster (see Figure 1 and Figure 2).

### Brain networks related to criticism

**Figure 3:**
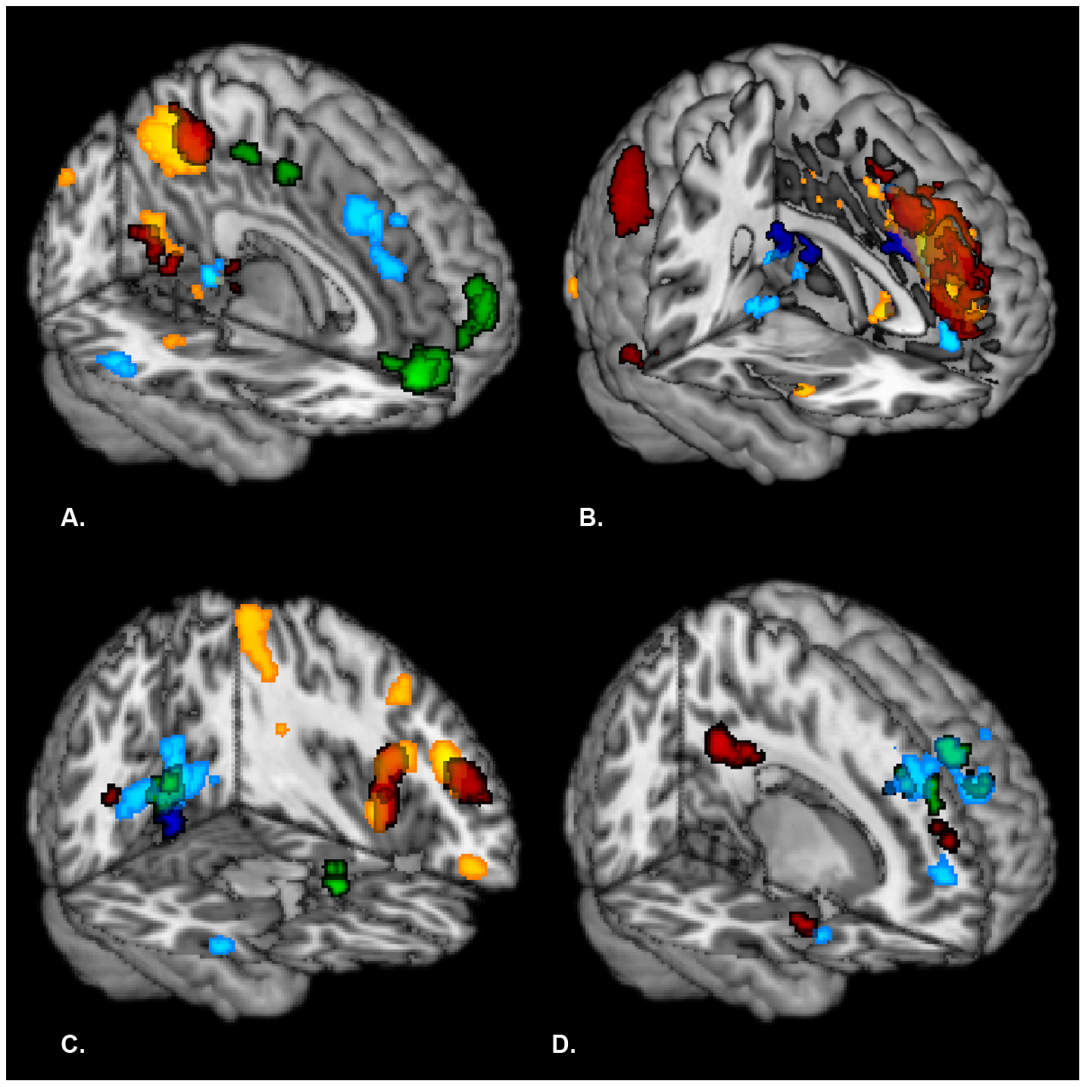
Functional connectivity patterns related to the thirteen seed regions overlayed on a MNI template for the different contrasts: (A) criticism > standard, (B) standard > criticism, (C) criticism > standard, positive correlation with neuroticism and (D) criticism > standard, negative correlation with neuroticism. Brain regions, showing enhanced functional connectivity to our thirteen seed regions, are depicted in red for seed regions that belong to the prefrontal cluster, in yellow for seed regions that belong to the fronto-temporal cluster, in green for seed regions that belong to the occipito-parietal cluster and in light blue for seed regions that belong to the amygdala/hippocampal cluster. Connectivity results for the seed regions anterior cingulate cortex and SFG(BA10) are depicted in dark blue. Results were corrected on FWE cluster level (k>20) with an initial threshold of p<0.001 uncorrected.

The criticism and standard session were contrasted for each of thirteen seed regions (see Figure 3 and Table 2). First, brain regions were identified that were functionally connected to the prefrontal cluster. When contrasting the criticism session and standard session, this cluster revealed enhanced functional connectivity with the precuneus, superior parietal gyrus, calcarine sulcus, lingual gyrus, fusiform gyrus, superior occipital gyrus and middle cingulate gyrus. The reverse contrast (standard > criticism) revealed increased functional connectivity between the prefrontal cluster and the superior medial frontal gyrus, superior frontal gyrus, anterior cingulate gyrus, middle cingulate gyrus, supplementary motor area, middle frontal gyrus, insula, inferior frontal gyrus, precentral gyrus, middle temporal gyrus, inferior temporal gyrus, inferior parietal gyrus, angular gyrus and supramarginal gyrus.

**Table 2:** Functional connectivity results related to criticism and associations with neuroticism.

Cluster	Seed region	Correlate	Cluster size	T-value	Z-value	Coordinate			
						x	y		z
Criticism versus standard									
1	Left superior frontal gyrus	Superior parietal gyrus/	87	4.83	4.30	−18	−64	44	
		Precuneus		3.70	3.43	−20	−62	34	
1*	Left superior frontal gyrus	Precuneus	303	4.82	4.30	10	−50	50	
				4.18	3.81	10	−42	48	
				4.14	3.78	0	−46	54	
1*	Left superior frontal gyrus	Calcarine sulcus	124	4.55	4.10	26	−60	12	
				4.43	4.01	22	−52	6	
				3.84	3.55	16	−64	20	
1*	Superior frontal gyrus (BA9)	Lingual gyrus/	172	5.45	4.74	−36	−52	−2	
		Fusiform gyrus		4.52	4.07	−16	−44	−6	
				4.49	4.05	−34	−60	−4	
1	Superior frontal gyrus (BA9)	Superior parietal gyrus/	103	5.25	4.60	−16	−66	46	
		Superior occipital gyrus/		4.29	3.90	−20	−64	38	
		Precuneus							
1*	Superior frontal gyrus (BA9)	Calcarine sulcus/	137	5.12	4.51	22	−52	6	
		Lingual gyrus		4.77	4.26	32	−64	16	
				4.48	4.04	26	−60	12	
1*	Superior frontal gyrus (BA9)	Precuneus/	338	5.02	4.44	0	−46	52	
		Middle cingulate gyrus		4.99	4.42	10	−44	52	
				3.80	3.52	−6	−52	56	
2*	Left inferior frontal gyrus	Precuneus	615	6.14	5.19	0	−56	50	
				4.88	4.34	10	−44	48	
				4.51	4.07	12	−58	46	
2	Left inferior frontal gyrus	Lingual gyrus/	99	4.64	4.16	14	−54	8	
		Calcarine sulcus/							
		Precuneus							
2	Left inferior frontal gyrus	Calcarine sulcus/	81	3.92	3.61	−2	−74	16	
		Lingual gyrus		3.85	3.56	6	−64	16	
				3.55	3.31	2	−60	8	
2*	Left insula	Precuneus	217	4.09	3.74	6	−62	48	
				3.88	3.58	14	−58	44	
				3.88	3.58	8	−52	42	
3*	Posterior cingulate gyrus/precuneus	Medial orbital frontal gyrus	133	6.30	5.29	2	50	−10	
3*	Cuneus	Medial orbital frontal gyrus	323	6.23	5.25	0	54	-10	
				5.37	4.68	0	46	−8	
				4.51	4.06	−−12	52	−6	
3*	Cuneus	Superior frontal gyrus/	158	5.36	4.68	−16	62	16	
		Superior medial frontal gyrus		3.98	3.65	−12	62	6	
4	Left amygdala	Superior medial frontal gyrus	125	4.23	3.85	−2	34	40	
				4.10	3.75	6	30	48	

Standard versus criticism								
1*	Left superior frontal gyrus	Superior medial frontal gyrus/	1381	6.22	5.24	−10	28	44
		Superior frontal gyrus/		6.21	5.23	10	46	42
		Anterior cingulate gyrus/		5.65	4.87	8	32	50
		Supplementary motor area						
1	Left superior frontal gyrus	Insula/Inferior frontal gyrus pars orbitalis	103	6.05	5.13	−28	18	−16
1*	Superior frontal gyrus (BA9)	Superior medial frontal gyrus	1841	6.90	5.65	−8	26	44
		Superior frontal gyrus/		6.35	5.32	4	32	50
		Anterior cingulate gyrus/		6.33	5.31	−6	42	38
		Middle cingulate gyrus/						
		Supplementary motor area						
1*	Superior frontal gyrus (BA9)	Middle frontal gyrus/	495	5.31	4.64	−48	22	36
		Inferior frontal gyrus pars triangularis/		5.21	4.57	−54	18	28
		Inferior frontal gyrus pars opercularis/		5.17	4.54	−48	10	46
		Precentral gyrus						
1	Superior frontal gyrus (BA9)	Middle temporal gyrus/	90	5.23	4.59	54	−40	−−10
		Inferior temporal gyrus						
1*	Superior frontal gyrus (BA9)	Superior frontal gyrus/	193	5.19	4.56	32	58	14
		Middle frontal gyrus		4.54	4.09	32	46	8
				4.02	3.69	42	56	8
1	Superior frontal gyrus (BA9)	Inferior frontal gyrus pars orbitalis/	82	5.18	4.55	−42	38	−4
		Inferior frontal gyrus pars triangularis						
1*	Superior frontal gyrus (BA9)	Inferior parietal gyrus/	261	4.98	4.41	54	−52	46
		Angular gyrus		4.47	4.04	44	−60	52
				4.41	3.99	52	−46	40
1	Superior frontal gyrus (BA9)	Middle temporal gyrus/	74	4.48	4.04	−58	−60	24
		Angular gyrus/		3.73	3.45	−54	−62	34
		Supramarginal gyrus/						
		Inferior parietal gyrus						
1	Superior frontal gyrus (BA9)	Middle frontal gyrus	112	4.35	3.95	38	20	50
				4.14	3.78	42	22	38
				3.88	3.58	50	24	40
2*	Left inferior frontal gyrus	Superior medial frontal gyrus/	986	6.39	5.35	−4	42	38
		Anterior cingulate gyrus/		6.10	5.16	0	34	40
		Middle cingulate gyrus/		5.12	4.51	−6	34	48
		Supplementary motor area						
2	Left inferior frontal gyrus	Inferior frontal gyrus pars opercularis	80	4.14	3.78	−48	16	0
		Inferior frontal gyrus pars triangularis						
		Insula						
2	Left insula	Insula	97	5.33	4.65	−42	12	2
		Inferior frontal gyrus pars opercularis						
2	Left insula	Middle cingulate gyrus/	117	4.80	4.28	6	20	34
		Supplementary motor area		4.27	3.88	4	10	48
				3.89	3.58	2	20	46
2*	Left temporal pole	Superior medial frontal gyrus/	784	6.17	5.21	−4	32	48
		Anterior cingulate gyrus		5.88	5.02	−6	46	28
				5.76	4.95	2	46	42
2	Left temporal pole	Inferior frontal gyrus pars opercularis/	105	4.90	4.35	−40	20	36
		Middle frontal gyrus		3.91	3.60	−40	12	42
4*	Right hippocampus	Hippocampus/	347	5.14	4.53	20	−36	6
		Lingual gyrus/		4.88	4.34	18	−46	0
		Calcarine sulcus		4.24	3.86	10	−62	8
1, 2*	Anterior cingulate gyrus	Anterior cingulate gyrus/	1072	7.02	5.72	−4	40	26
		Superior medial frontal gyrus/		6.20	5.23	−4	28	32
		Middle cingulate gyrus/		6.00	5.10	16	44	36
		Middle frontal gyrus						
1, 2	Anterior cingulate gyrus	Medial orbital frontal gyrus	71	4.80	4.28	−8	58	0
				3.82	3.53	−6	60	−8
1, 2*	Superior frontal gyrus (BA10)	Anterior cingulate gyrus/	650	5.85	5.01	−8	40	26
		Superior medial frontal gyrus/		5.67	4.89	2	40	30
		Superior frontal gyrus		5.23	4.59	18	48	36

Criticism versus standard, positive correlation with neuroticism								
1	Left superior frontal gyrus	Middle frontal gyrus/	108	4.84	4.31	−44	42	18
		Inferior frontal gyrus pars triangularis		3.89	3.59	−38	36	20
				3.44	3.22	−44	30	26
1	Superior frontal gyrus (BA9)	Supplementary motor area	104	5.03	4.45	10	0	50
1	Superior frontal gyrus (BA9)	Middle frontal gyrus/	79	4.62	4.15	−36	36	26
		Inferior frontal gyrus pars triangularis		3.61	3.36	−44	42	20
1*	Superior frontal gyrus (BA9)	Precentral gyrus/	147	4.59	4.13	−40	6	20
		Inferior frontal gyrus pars opercularis		4.52	4.07	−42	2	6
		Insula						
		Rolandic operculum						
2*	Left inferior frontal gyrus	Inferior frontal gyrus pars triangularis/	227	6.35	5.32	−42	28	28
		Inferior frontal gyrus pars opercularis/		4.25	3.87	−32	18	24
		Precentral gyrus/		4.04	3.70	−38	12	28
		Rolandic operculum						
2	Left inferior frontal gyrus	Inferior frontal gyrus pars orbitalis/	99	4.50	4.06	46	36	−8
		Middle frontal gyrus		4.49	4.05	48	24	−12
				4.23	3.85	40	42	14
2	Left inferior frontal gyrus	Inferior parietal gyrus/	95	4.39	3.97	−40	−60	56
		Angular gyrus		3.92	3.61	−40	−54	40
2	Left insula	Middle frontal gyrus	71	5.24	4.59	36	40	18
				4.27	3.88	28	42	16
2	Left insula	Inferior parietal gyrus	106	5.09	4.49	−50	−34	44
3	Posterior cingulate gyrus/precuneus	Cuneus	93	5.11	4.50	−4	−88	28
				4.66	4.18	−6	−92	18
3*	Cuneus	Calcarine sulcus/	271	4.91	4.36	−4	−82	−4
		Lingual gyrus		4.89	4.34	−6	−68	6
				4.33	3.93	−10	−74	0
3	Cuneus	Inferior frontal gyrus pars triangularis/	74	4.09	3.75	46	14	22
		Inferior frontal gyrus pars opercularis		3.79	3.51	50	20	30
4	Left amygdala	Lingual gyrus/	118	5.15	4.53	−6	−64	2
		Calcarine sulcus		4.51	4.07	−6	−66	12
				3.75	3.47	−6	−54	6
4*	Left hippocampus	Lingual gyrus/	525	5.70	4.91	−14	−76	−8
		Calcarine sulcus		5.64	4.87	−18	−70	−2
				4.83	4.30	−6	−68	2
4	Left hippocampus	Lingual gyrus/	114	5.06	4.46	14	−62	4
		Calcarine sulcus		3.80	3.51	26	−60	2
4*	Left hippocampus	Cuneus/	197	5.05	4.46	−4	−86	24
		Superior occipital gyrus/		4.37	3.96	−14	−88	24
		Calcarine sulcus		3.99	3.66	4	−90	10
4	Left hippocampus	Superior occipital gyrus	86	4.78	4.27	24	−78	20
				4.17	3.81	20	−88	20
1, 2	Superior frontal gyrus (BA10)	Postcentral gyrus/	115	4.83	4.30	48	−16	52
		Precentral gyrus		4.47	4.04	42	−14	58

Criticism versus standard, negative correlation with neuroticism								
1	Left superior frontal gyrus	Middle temporal gyrus/	66	5.36	4.68	−38	−58	20
		Angular gyrus						
1	Left superior frontal gyrus	Middle temporal gyrus/	75	4.57	4.11	−50	4	−18
		Superior temporal pole		4.11	3.76	−50	12	−14
1	Left superior frontal gyrus	Posterior cingulate gyrus	66	4.48	4.05	−10	−32	30
				4.05	3.71	−8	−44	24
				3.73	3.46	−16	−38	28
1*	Superior frontal gyrus (BA9)	Posterior cingulate gyrus	238	5.03	4.45	−10	−28	32
				4.59	4.13	12	−46	28
				4.56	4.10	−8	−44	24
1	Superior frontal gyrus (BA9)	Angular gyrus/	76	4.02	3.69	50	−56	28
		Superior temporal gyrus		3.35	3.15	50	−58	36
3	Posterior cingulate gyrus/precuneus	Postcentral gyrus/	83	5.84	5.00	54	−2	22
		Rolandic operculum						
3	Posterior cingulate gyrus/precuneus	Middle cingulate gyrus	83	5.41	4.71	−4	24	36
				4.30	3.90	4	26	34
3*	Cuneus	Insula	161	6.48	5.40	−42	14	−12
				3.67	3.41	−30	18	−12
				3.46	3.24	−42	16	0
4*	Left amygdala	Superior medial frontal gyrus/	162	5.04	4.46	6	36	40
		Middle cingulate gyrus		4.22	3.85	−8	38	42
				4.12	3.77	8	28	36
4	Left hippocampus	Superior medial frontal gryus/	117	4.44	4.02	−10	40	48
		Superior frontal gyrus		4.43	4.01	−20	18	50
				4.34	3.93	−16	30	48
4	Left hippocampus	Superior frontal gyrus/	124	4.39	3.98	16	52	34
		Superior medial frontal gyrus		4.16	3.80	20	42	36
				3.87	3.57	10	48	42
4	Right hippocampus	Middle frontal gyrus	96	4.99	4.41	26	32	20
				4.01	3.68	42	32	40
				3.82	3.53	36	26	36
1, 2	Anterior cingulate gyrus	Calcarine sulcus/	96	4.50	4.06	−8	−64	10
		Lingual gyrus		3.62	3.37	−10	−52	2
1, 2*	Superior frontal gyrus (BA10)	Angular gyrus	129	4.73	4.23	52	−50	34
				4.24	3.86	46	−62	28
				4.00	3.67	48	−54	24

Peak activations with corresponding T-values and Z-values of brain regions, which showed enhanced functional connectivity to our selected seed regions per cluster for the contrasts (criticism > standard), (standard > criticism) and (criticism > standard x neuroticism). Results were corrected on FWE cluster level (k>20) with an initial threshold of p<0.001 uncorrected. An asterisk (*) was used to denote clusters that survived multiple comparisons correction for applying thirteen seed regions (FWE cluster level (k>20) p = 0.05/13 = p<0.003).

Second, the functional connectivity pattern was determined for the fronto-temporal cluster. For criticism compared to standard, this cluster showed stronger functional connectivity with the precuneus, lingual gyrus and calcarine sulcus. When standard was contrasted with criticism, enhanced functional coupling was found between the fronto-temporal cluster and the superior medial frontal gyrus, anterior cingulate gyrus, middle cingulate gyrus, supplementary motor area, middle frontal gyrus, insula and inferior frontal gyrus.

Third, brain areas were identified that were functionally connected to the occipito-parietal cluster. When criticism was contrasted with standard, the occipito-parietal cluster showed stronger functional connections with the medial orbital frontal gyrus. For standard compared to criticism, no significant results were found.

Finally, the functional connectivity pattern was identified for the amygdala/hippocampal cluster. The contrast (criticism > standard) showed enhanced functional coupling between this cluster and the superior medial frontal gyrus. The reverse contrast (standard > criticism) revealed increased functional connectivity between the amygdala/hippocampal cluster and the hippocampus, lingual gyrus and calcarine sulcus.

### The effect of neuroticism on criticism-related brain networks

Interactions between criticism-related functional connectivity and neuroticism were investigated by calculating positive as well as negative correlations with neuroticism for the contrast (criticism > standard) per seed region (see Figure 3 and Table 2).

First, we identified the functional connectivity pattern for the prefrontal cluster that was modulated by neuroticism. Neuroticism correlated positively with functional connectivity between this cluster and the middle frontal gyrus, supplementary motor area, inferior frontal gyrus, precentral gyrus, insula and rolandic operculum. Furthermore, neuroticism was negatively related to functional connectivity between the prefrontal cluster and the posterior cingulate gyrus, angular gyrus, superior temporal gyrus, middle temporal gyrus and superior temporal pole.

Second, the functional connectivity pattern was identified for the fronto-temporal cluster on which neuroticism had a modulatory effect. Neuroticism showed a positive correlation with functional connectivity between this cluster and the middle frontal gyrus, inferior parietal gyrus, angular gyrus, inferior frontal gyrus, precentral gyrus and rolandic operculum. No significant functional connectivity results were found, when a negative correlation was calculated with neuroticism.

Third, brain areas were determined for which their functional connection with the occipito-parietal cluster was modulated by neuroticism. Neuroticism was positively associated with functional connectivity between this cluster and the cuneus, calcarine sulcus, lingual gyrus and inferior frontal gyrus. Furthermore, neuroticism correlated negatively with functional connectivity between the occipito-parietal cluster and the middle cingulate gyrus, insula, rolandic operculum and postcentral gyrus. Lastly, we identified the functional connectivity pattern for the amygdala/hippocampal cluster on which neuroticism had a modulatory effect. Neuroticism revealed a positive correlation with functional connectivity between this cluster and the lingual gyrus, calcarine sulcus, superior occipital gyrus and cuneus. Furthermore, neuroticism was negatively related to functional connectivity between the amygdala/hippocampal cluster and the superior medial frontal gyrus, superior frontal gyrus, middle frontal gyrus and middle cingulate gyrus.

## Discussion

In the current study, we developed a novel resting-state paradigm to investigate the effect of criticism on functional brain connectivity and associations with neuroticism. The cluster analysis revealed four clusters based on selected seed regions related to self-reflective processing and stress-regulation. During the processing of criticism, these clusters showed enhanced functional connectivity with brain areas involved in emotion processing and social cognition, while they showed reduced connectivity with brain regions related to the default mode network and higher-order cognitive control. Furthermore, the findings revealed that neuroticism modulated functional connectivity between aforementioned clusters and brain areas associated with the appraisal, expression and regulation of negative emotions.

### Brain networks related to criticism

First, decoupling was found between the prefrontal and fronto-temporal cluster and brain areas related to the default mode network during the processing of criticism. The default state of the brain is supported by a distributed network of anterior and posterior cortical midline structures, the lateral parietal cortex and hippocampal formation [29]. Activity in this network has been observed during passive experimental control conditions and is involved in self-relevant internal cognitive processes [29]. Our finding may suggest that individuals were more externally oriented during the criticism session than during the standard session. Furthermore, the prefrontal and fronto-temporal cluster displayed reduced functional connectivity with several prefrontal brain regions as well. This finding is in line with previous research showing that even mild acute uncontrollable stressors are able to disrupt prefrontal functioning [30], [31]. However, the effects of stress on the brain are not always disadvantageous. Emotional stress can bias processing in favor of a salient stimulus that is relevant to the individuals' current situation [30], [31]. In the present paradigm, the salient stimulus took the form of criticism that was expressed onto the subjects' behavior in the scanner. Accordingly, we found enhanced functional coupling between the clustered seed regions and brain areas involved in emotion processing and social cognition during the processing of criticism. Our results fit with the integrative model of emotion understanding proposed by Spunt and Lieberman (2012) [32]. The authors suggested that first, the mirror neuron system is recruited during the identification of behavior and subsequently, the mentalizing system is recruited in order to make a causal attribution to the observed behavior [32]–[34].

In line with the first part of Spunt and Lieberman's model (2012), we found enhanced functional coupling between the fronto-temporal cluster (specifically the inferior frontal gyrus, IFG) and a number of parietal regions, specifically the precuneus [32]. Previous research has shown that the IFG possesses mirror neuron properties [35], [36] and that it is involved in the identification of emotional prosody by utilizing motor representations with regard to the production of a given intonation [37]–[40]. Such sensorimotor patterns may facilitate the identification of other people's feelings by simulating their mental state [37], [38]. This step precedes the mental process of mentalizing in which emotions are attributed to social causes [32]. One of the connections through which both systems are integrated is the connection between the IFG and precuneus (the latter structure is an integral part of the mentalizing system) [32]. This finding is in line with our results, except that Spunt et al. (2012) found the right IFG to be connected to the precuneus instead of the left [32]. However, this distinction might be explained by a difference in task paradigm. In the paradigm of Spunt and Lieberman (2012), participants were instructed to infer an individuals' emotional state from motor behavior in contrast to linguistic input [32]. In accord, a recent meta-analysis on the diversity of the inferior frontal gyrus revealed that movement control could be attributed to the right hemisphere, while functions related to empathy, language and working memory could be attributed to the left hemisphere [35].

Alternatively, a connection between the left IFG and precuneus has been implicated in the recollection of personal episodes from the past (autobiographical memory) [41]. There is evidence linking autobiographical memory to social cognition by showing a common neural substrate for both mental processes, including the inferior frontal gyrus and precuneus/posterior cingulate gyrus [42]. This functional overlap might promote the construction of predictions regarding other people's feelings and behavior by drawing upon personal past experiences [42].

With regard to the second part of Spunt and Lieberman's model (2012), we found enhanced functional connectivity between the prefrontal cluster and several parietal regions (including the precuneus and superior parietal gyrus) and the parietal cluster and medial orbital frontal cortex (OFC) [32]. These regions have been implicated in mentalizing and represent the cognitive and affective components of Theory of Mind (ToM), respectively [33], [43]–[45]. The dorsal medial prefrontal cortex (dmPFC, overlapping with the prefrontal cluster) is involved in inferring what other people *think*, while the ventral medial prefrontal cortex (vmPFC, overlapping with the medial OFC) is implicated in making inferences about what other people *feel* [33]. Both components are indirectly connected to the precuneus/posterior cingulate gyrus in the higher association cortex, which is engaged in self-referential processing [43], [46]. Furthermore, a connection has been found between the dmPFC and precuneus during the assessment of social relationships and their implications [47] and autobiographical memory [46], [48]. Moreover, the orbital frontal cortex has been associated with decoding mental states by extracting social information from the environment, such as an individuals' tone of voice [49].

Finally, we found that the left amygdala coactivated with the dmPFC during the processing of criticism. This finding is consistent with the postulated framework of Etkin et al. (2011), in which a positive connection between abovementioned brain regions is attributed to the appraisal and expression of negative emotions [50]. Furthermore, various studies have shown the dmPFC and amygdala to be part of a network underlying emotion regulation [51], [52].

### The effect of neuroticism on criticism-related brain networks

Enhanced functional coupling was found between the prefrontal and fronto-temporal cluster and the lateral prefrontal cortex (LPFC) in individuals scoring higher on neuroticism during the processing of criticism. This region -among others- is involved in the cognitive control over negative emotions [51], specifically during cognitive reappraisal [51], [53], [54]. Reappraisal can be defined as a strategy in which individuals explicitly regulate their emotions by reinterpreting the meaning of an affective stimulus to reduce its emotional impact [51]. Individual differences in the capacity to employ cognitive control in response to emotionally distressing experiences have been related to variation in adaptive functioning. The impact that these experiences ultimately have on well-being are determined by regulatory success [51]. Generally, high neurotic individuals cope poorly with daily hassles and frequently experience mood spillovers [3], [4]. Furthermore, fMRI studies systematically showed that high neurotic individuals are more sensitive to a wide range of negative emotional stimuli, e.g. sad, angry and fearful faces; negative and arousing scenes; negative words; and aversive anticipatory cues [55]–[62]. In addition, high neurotic individuals are more self-critical [9] and are overly sensitive to criticism by others [5]. These findings and ours may indicate that individuals scoring higher on neuroticism need greater regulatory efforts in order to gain cognitive control over their emotions. However, caution is needed since other functional roles of the LPFC cannot be ruled out [18].

Furthermore, we found decreased functional connectivity between the prefrontal cluster and several default mode brain regions in individuals scoring higher on neuroticism during the processing of criticism. As described before, the default mode network has been related to processes such as self-related processing, mental simulation, introspection, future planning and emotion regulation [29], [63]. This finding indicates that although frontal connections are strengthened in high neurotic individuals during the processing of criticism, multiple other long range connections -important for regulating negative emotions- are weakened. It seems that the aforementioned frontal circuit may play a compensatory role by increasing its functional connectivity. Previous research has shown that patients with anxiety disorders also demonstrate decreased default mode functioning in comparison to healthy controls, when they are not given explicit instructions on how to regulate their emotions [63]. In addition, decreased functional coupling was found between the amygdala/hippocampal cluster and a number of frontal regions, including the dmPFC and dorsal lateral prefrontal cortex (dlPFC) in individuals scoring higher on neuroticism during the processing of criticism. As previously mentioned, a connection between these brain areas is involved in the appraisal and expression of negative emotions [50]. It seems that multiple aspects of emotion processing are affected in high neurotic individuals during the processing of criticism, which may increase their sensitivity to negative social-evaluation.

### Limitations

Several limiting factors can be mentioned with regard to the current study. First, a seed-based functional connectivity method was used to quantify connections within the brain. Since this is a correlation based method, we cannot distinguish between direct or indirect pathways between brain regions or assess causal directions between them. Second, a difference in acquisition parameters existed between the two resting-state sessions. The influence of such a difference on functional connectivity has been investigated by van Dijk et al. (2010). In their study, temporal (TR 2.5 versus 5) as well as spatial (voxel size 2 mm^3^ versus 3 mm^3^) resolution were varied between runs. The authors concluded that these factors have a minimal effect on functional connectivity measures [22]. Notably, the differences in TR and voxel size were much smaller in the current study (TR 2 versus 2.29 and voxel size 3.2×3.2×2.5 versus 3.44×3.44×3). Therefore, we deem it unlikely that differences in acquisition parameters biased our results substantially. Specifically, the functional connectivity findings related to neuroticism cannot be explained by differential acquisition parameters, since all participants were scanned using the same protocol. Third, a test-retest effect (i.e. time on task) could not be examined in the current study. An option would have been to present neutral comments between the two runs to half of the subjects, however this would have doubled the sample size. Alternatively, counter balancing task order is often applied to disentangle task effects from effects related to test-retest. Note that this was not option because the temporal dynamics of the manipulation are unknown. Investigating the whole-brain functional connectivity dynamics as a consequence of the manipulation would be particularly interesting and should improve the sensitivity of the analysis even further. Future research may benefit from studying such time-varying aspects in functional connectivity, for instance, to elucidate how long changes in brain networks related to negative affect persist and whether this pattern is different for high and low neurotic individuals. However, we need to emphasize that having a fixed task order puts constraints on the interpretation of our results. In principle, the findings could be explained by factors such as habituation effects. Nonetheless, differences were found between the two runs that correlated with neuroticism. It is improbable that high neurotic individuals would have reacted in a similar manner to neutral comments, since it is a robust finding in neuroticism research that these individuals express heightened emotional reactivity to negative events [55]–[62] or react differently to prolonged scan duration. Fourth, no objective stress measures were assessed during the experiment (e.g. heart rate, respiration and cortisol) in order to perform a manipulation check and verify that receiving criticism is indeed experienced as a stressful and arousing event. Nevertheless, the current paradigm has never been used before and now that it has shown significant effects, it can be investigated more extensively with accompanying measures.

## Conclusion

In the current study, we used a novel resting-state paradigm to investigate the effect of criticism on functional brain connectivity and associations with neuroticism. The findings showed that brain regions involved in emotion processing and social cognition were recruited during the processing of criticism, while default mode activity and higher-order cognitive control functions were attenuated. These results may suggest that the criticized person is attempting to understand the beliefs, perceptions, emotions and goals of the critic in order to facilitate flexible and adaptive social behavior. Furthermore, individuals scoring higher on neuroticism showed alterations in functional connectivity between brain areas involved in the appraisal, expression and regulation of negative emotions. These results underscore the general emotional liability that characterizes high neurotic individuals and provide insights into the underlying neurobiological mechanisms that predispose such individuals to the development of mood disorders.

## Supporting Information

File S1 **Supplementary material.**
(DOCX**)**
