# Supplementary material for: The Effect of Criticism on Functional Brain Connectivity and Associations with Neuroticism
Source: PLoS One. 2013 Jul 26;8(7):e69606. doi: 10.1371/journal.pone.0069606 (PMC3724923; doi:10.1371/journal.pone.0069606)
Supplement: File S1 — Supplementary material. (DOCX) [file pone.0069606.s001.docx]

# S1 Supplementary material

## Stimulus pilot

A pilot study was conducted to validate whether the tone of the three critical remarks was indeed perceived as increasingly agitated and whether the receipt of these remarks was indeed experienced as negative, stressful and arousing. The critical remarks were presented in a random order to ten students (5 women, age range 18-30 years) of the University of Groningen. Participants were instructed to order the critical remarks from least agitating to most agitating. All participants ordered the critical remarks as expected (remark 1 as least agitating and remark 3 as most agitating). Except for one participant, who rated remark 2 more agitating than remark 3. A separate pilot study was conducted with ten different students (5 women, age range 18-30 years) from the University of Groningen to test the effect of the critical remarks on mood. Participants were asked to fill in the Positive Affect/Negative Affect Scale (PANAS), once before the aural presentation of the critical remarks and once after. This was asked to participants who just came out of the scanner for another experiment, so they could imagine the situation. Positive affect significantly decreased (T_(9)_= 2.85, p<0.05) after the presentation of the critical remarks, while negative affect significantly increased (T_(9)_= -4.59, p<0.05).

## Clustering analysis


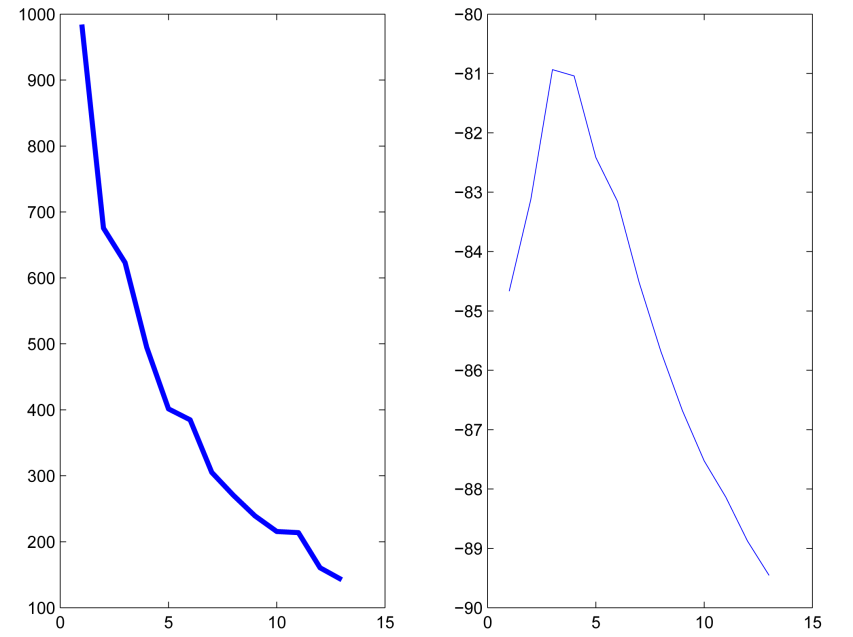


Figure S1 The number of clusters was estimated by creating Cattell’s screeplot (left) and a maximum profile log-likelihood (right) based on the extracted eigenvalues of the covariance matrix of D. Matrix D resulted from concatenating the connectivity maps of the thirteen seed regions for the contrast (criticism > standard) averaged over subjects. The first four principal components could be retained.


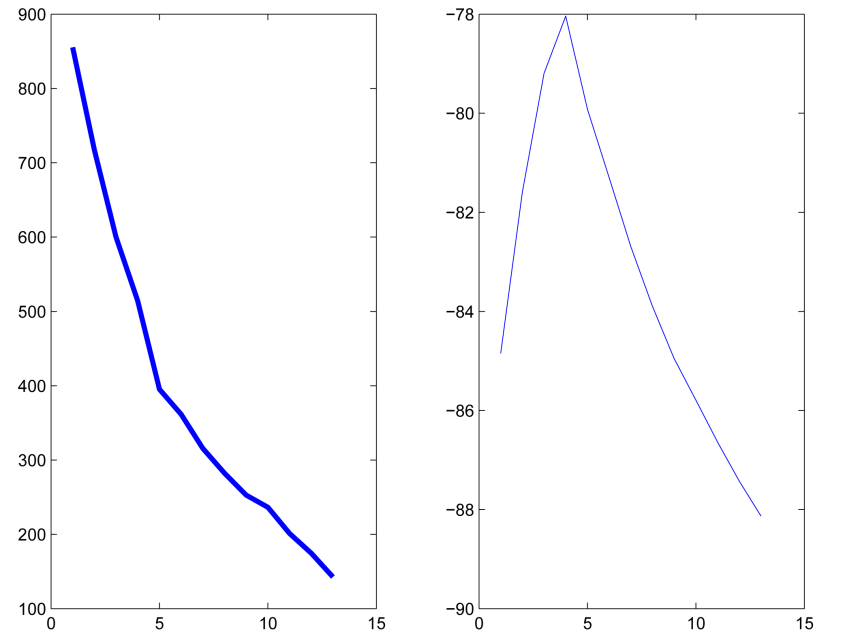


Figure S2a The number of clusters was estimated by creating Cattell’s screeplot (left) and a maximum profile log-likelihood (right) based on the extracted eigenvalues of the covariance matrix of D. Matrix D resulted from concatenating the connectivity maps of the thirteen seed regions for the contrast (criticism > standard x neuroticism) averaged over subjects. The same four component solution was found as for the contrast (criticism > standard) (see 2. Clustering analysis, Figure S1 in File S1).


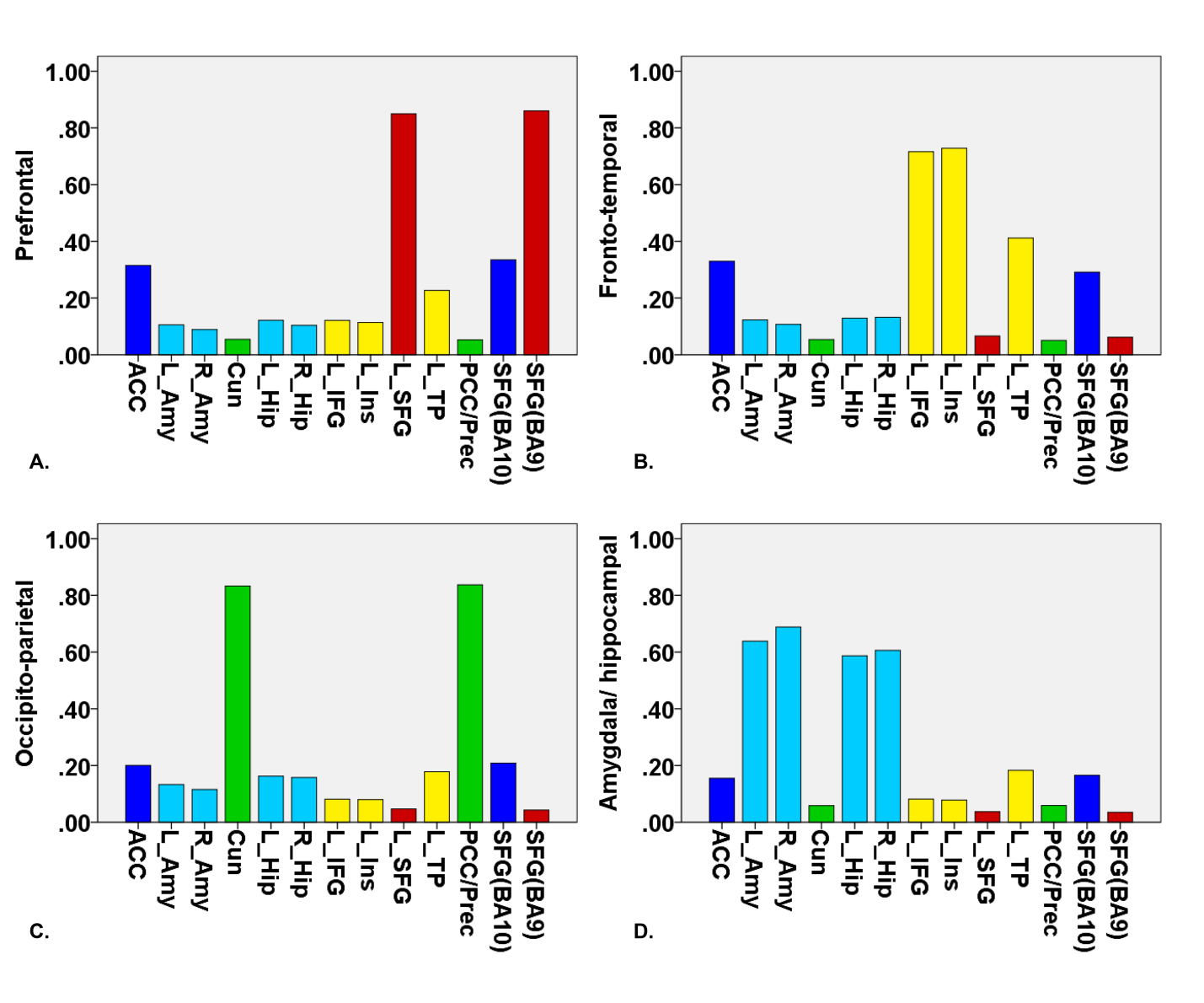


Figure S2b The same cluster partition was found for the contrast (criticism > standard x neuroticism) as for the contrast (criticism > standard) (see Figure 1 in the main text). (A) prefrontal cluster (red bars), (B) fronto-temporal cluster (yellow bars), (C) occipito-parietal cluster (green bars) and (D) amygdala/hippocampal cluster (light blue bars). The seed regions anterior cingulate cortex and SFG(BA10) are depicted in dark blue. On the x-axis, the different seed regions can be found in alphabetical order. On the y-axis, membership degrees are continuously expressed as proximities to a cluster centroid, containing values between 0 and 1. ACC, anterior cingulate cortex; L_Amy, left amygdala; R_Amy, right amygdala; Cun, cuneus; L_Hip, left hippocampus; R_Hip, right hippocampus; L_IFG, left inferior frontal gyrus; L_Ins, left insula; L_SFG, left superior frontal gyrus; L_TP, left temporal pole; PCC/Prec, posterior cingulate cortex/precuneus; SFG(BA10), superior frontal gyrus (BA10); SFG(BA9), superior frontal gyrus (BA9).
